# Virulence genes and phylogenetic analysis of antibiotic-resistant *Escherichia coli* isolated from pig slaughterhouses in Banten Province, Indonesia

**DOI:** 10.14202/vetworld.2025.1242-1252

**Published:** 2025-05-21

**Authors:** Hadri Latif, Debby Fadhilah Pazra, Chaerul Basri, Dinda Iryawati, I. Wayan Teguh Wibawan, Puji Rahayu

**Affiliations:** 1 Division of Veterinary Public Health and Epidemiology, School of Veterinary Medicine and Biomedical Sciences, IPB University, Bogor 16680, Indonesia; 2 Division of Animal Health, Bogor Agricultural Development Polytechnic, Bogor 16730, Indonesia; 3 Division of Public Health and Ethicomedicolegal, Faculty of Medicine, IPB University, Bogor 16680, Indonesia; 4 Division of Medical Microbiology, School of Veterinary Medicine and Biomedical Sciences, IPB University, Bogor 16680, Indonesia; 5 Quality Control Laboratory and Certification of Animal Products, Bogor 16161, Indonesia

**Keywords:** antimicrobial resistance, *Escherichia coli*, pathotype diversity, phylogenetics, pig slaughterhouses, virulence genes, zoonoses

## Abstract

**Background and Aim:**

*Escherichia coli* is a prominent zoonotic pathogen with diverse virulence factors and significant antibiotic resistance, particularly in pig farming environments. Pig slaughterhouses are critical points of potential bacterial transmission to humans and the environment. Comprehensive genomic surveillance of *E. coli* in these settings remains limited in Indonesia. This study aimed to investigate the phylogenetic distribution, virulence gene profiles, pathotypes, and antibiotic resistance characteristics of *E. coli* isolated from pig slaughterhouses in Banten Province, Indonesia, using whole-genome sequencing.

**Materials and Methods:**

Environmental samples, including effluent and floor swabs (n = 200), were collected from 10 pig slaughterhouses. *E. coli* isolates were identified and previously characterized for antibiotic resistance. Genomic DNA was extracted and sequenced using the Oxford Nanopore MinION platform. Bioinformatic analyses, including virulence gene detection (VirulenceFinder), phylogenetic reconstruction (RAxML), and phylogroup determination (Clermont method), were conducted to classify isolates based on pathotype and genetic lineage.

**Results:**

Fifty-seven virulence genes were identified, including 46 associated with enteric pathotypes (Enterohemorrhagic *E. coli*: 35%, enterotoxigenic *E. coli*: 15%, and enteropathogenic *E. coli*: 5%) and 15 linked to extraintestinal pathotypes (uropathogenic *E. coli*: 95%, and neonatal meningitis *E. coli*: 5%). Phylogenetic analysis revealed five phylogroups - A, B1, D, G, and clade I - with A and B1 predominating. Most isolates (60%) exhibited a single pathotype, while a minority (5%) carried genes from multiple pathotypes. Serotypes O73, O78, and O157 were identified, with O73 being the most prevalent. No strong correlation was observed between phylogenetic clustering and virulence gene pathotype.

**Conclusion:**

The high prevalence of multidrug-resistant *E. coli* with diverse virulence genes in pig slaughterhouses highlights significant zoonotic and environmental health risks. These findings underscore the need for enhanced hygiene practices, antimicrobial stewardship, and longitudinal genomic surveillance in Indonesian pig production systems.

## INTRODUCTION

*Escherichia coli* is a widespread microorganism exhibiting exceptional adaptability across various ecological niches. It typically resides in the gastrointestinal tracts of healthy humans and animals as a commensal bacterium [1]. Nevertheless, certain *E. coli* strains are capable of causing a broad spectrum of diseases, ranging from gastrointestinal disorders to extraintestinal and systemic infections extraintestinal pathogenic *E. coli* (ExPEC) in both humans and animals [2]. Research has demonstrated that pathogenic *E. coli* strains may evolve from commensal ancestors through the acquisition of chromosomal or plasmid-borne virulence gene operons [3]. Diarrheagenic *E. coli* strains are categorized into six well-defined pathotypes based on their pathogenic characteristics: Enteropathogenic *E. coli* (EPEC), enterotoxigenic *E. coli* (ETEC), Enterohemorrhagic *E. coli* or Shiga toxin-producing *E. coli* (EHEC/STEC), enteroaggregative *E. coli* (EAEC), enteroinvasive *E. coli*, and diffusely adherent *E. coli* [4]. In addition, *E. coli* is a major etiological agent of urinary tract infections (UTIs), which include uropathogenic *E. coli* (UPEC), sepsis-associated *E. coli*, and neonatal meningitis-causing strains neonatal meningitis *E. coli* (NMEC) [5].

*E. coli* has been linked to colibacillosis, a common infectious disease affecting both swine and humans [6]. In swine, colibacillosis presents a major challenge to the pig industry due to its association with elevated morbidity and mortality rates [7]. ETEC, EPEC, and STEC are the principal agents responsible for neonatal Diarrhea, post-weaning Diarrhea, and edema disease in pigs [8], affecting nearly every phase of pig production. These pathotypes represent significant public health concerns as foodborne pathogens and have been implicated in fatal outbreaks worldwide [9, 10]. Pigs are widely recognized as important reservoirs of pathogenic *E. coli*, with the potential to contaminate pork products and transmit infections to humans [7].

Enteric *E. coli* pathotypes contribute to disease through distinct pathogenic mechanisms. Pathogenesis involves the expression of specific virulence factor genes that enable the organism to colonize, invade, and damage host tissues [11, 12]. A wide array of virulence factors related to both enteric and extraintestinal infections caused by *E. coli* has been identified in recent years [13]. Gaining insights into these virulence factors and the genes encoding them enhances our understanding of host-pathogen interactions at the molecular level, thereby informing the development of targeted prevention strategies [14].

Recent phylogenetic analyses have classified *E. coli* strains into eight phylogroups, including four primary groups (A, B1, B2, and D) and four additional groups (C, E, F, and Clade I) [15]. Mosquito *et al*. [16] have reported that commensal strains typically belong to phylogroups A and B1, while extraintestinal strains are more commonly associated with phylogroups B2 and D. Notably, our examination of *E. coli* isolates from Indonesian slaughterhouses identified unique combinations of virulence genes, including some that have not been previously reported, thereby emphasizing the genetic diversity and pathogenic potential of these strains. Thus, characterizing the distribution of phylogroups among *E. coli* isolates contributes valuable epidemiological insights and has significant implications for understanding transmission dynamics and informing public health strategies.

The extensive use of antibiotics in pig production for therapeutic, preventive, and growth-promoting purposes has played a key role in the emergence of multidrug-resistant (MDR) *E. coli* strains. Multiple studies have reported rising resistance to several classes of antibiotics, likely driven by excessive antibiotic application in swine production systems. This trend has contributed to treatment failures, clinical complications, and heightened rates of morbidity and mortality [17]. Prolonged antibiotic exposure is strongly correlated with increased resistance in pathogenic *E. coli* compared to commensal strains, underscoring serious threats to food safety and public health [18, 19].

Despite growing concerns regarding the public health implications of antibiotic-resistant *E. col*i in food-producing animals, there remains a paucity of comprehensive genomic investigations focusing on virulence gene profiles and phylogenetic lineages of *E. coli* in pig slaughterhouse environments in Indonesia. Most existing studies in the region have relied on phenotypic characterization and conventional molecular methods, which offer limited resolution in detecting pathogenic potential and genomic diversity. Moreover, there is insufficient knowledge about the co-occurrence of multiple pathotypes, the relationship between virulence gene carriage and phylogroups, and the possible environmental dissemination routes of MDR *E. coli* strains. This lack of whole-genome-based surveillance data hampers effective risk assessment, disease control, and public health interventions tailored to the pig production sector in Indonesia.

This study aimed to perform a comprehensive genomic analysis of *E. coli* isolates obtained from environmental samples in pig slaughterhouses across Banten Province, Indonesia. Specifically, the study sought to (i) identify virulence genes associated with enteric and extraintestinal pathotypes, (ii) classify isolates into phylogenetic groups and serotypes, (iii) assess the distribution of pathotypes and their relationship with phylogenetic clusters, and (iv) evaluate the public health significance of MDR *E. coli* in slaughterhouse environments. By employing whole-genome sequencing (WGS), this investigation contributes novel insights into the molecular epidemiology of pathogenic *E. coli* in a high-risk setting and underscores the need for improved biosecurity and surveillance systems.

## MATERIALS AND METHODS

### Ethical approval

Approval from the Institutional Animal Care and Use Committee was not required for this study, as sampling procedures were non-invasive and limited to environmental collection. Nevertheless, all samples were collected in strict accordance with the World Health Organization (WHO) Global Tricycle Surveillance guidelines, thereby ensuring methodological rigor and compliance with international standards. Specifically, the sampling followed the standardized procedures outlined in the WHO’s Global Tricycle Surveillance guidelines for extended-spectrum beta-lactamase (ESBL)-producing *E. coli* [20].

### Study period and location

The study was conducted from June to November 2024 in Banten and West Java Provinces, Indonesia.

### Sample collection and preparation

**Figure 1:**
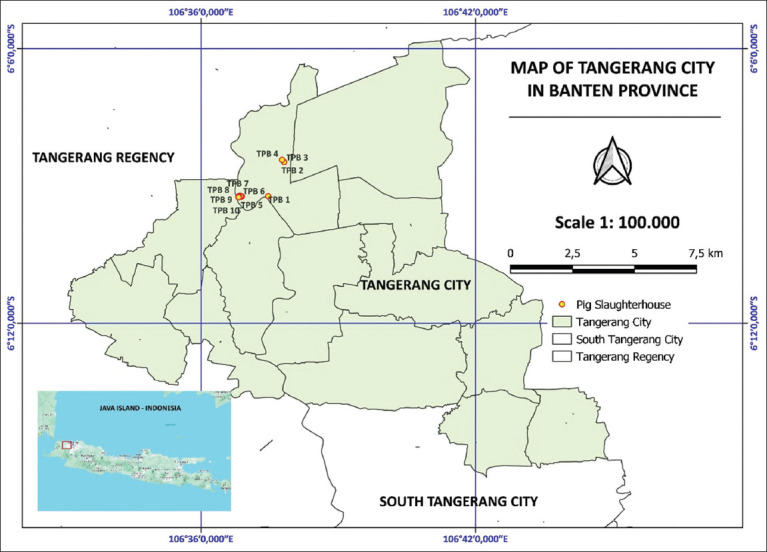
Pig slaughterhouse distribution in Banten province, Java, Indonesia. TPB=Tempat pemotongan babi (Pig Slaughterhouse) [Source: Map generated by QGIS 2.40.2 software].

This investigation included all 10 operational pig slaughterhouses in Banten Province, representing the first comprehensive study of its kind in the region. From each slaughterhouse, 20 samples were collected – 10 effluent samples (designated as isolate E) and 10 floor swab samples (designated as isolate F). The geographic distribution of the sampling locations is illustrated in Figure 1. The isolates used in this study had been previously obtained and characterized, facilitating a more in-depth genomic investigation with a focus on antibiotic resistance and virulence genes, thereby extending earlier microbiological research [21]. Isolation and identification of *E. coli* were conducted in accordance with the WHO Global Tricycle Surveillance ESBL *E. coli* guidelines [20]. In addition, antimicrobial susceptibility testing of the isolates had been carried out in prior work [22]. This testing employed the Kirby–Bauer disk diffusion method on Mueller–Hinton Agar, in compliance with the Clinical and Laboratory Standards Institute guidelines published in 2018 [23].

### DNA extraction and quality control

Genomic DNA (gDNA) was extracted from the *E. coli* isolates using the PowerWater DNA Extraction Kit, following the manufacturer’s protocol without deviation (Qiagen, Hilden, Germany). DNA concentration and purity were assessed using a Qubit fluorometer to ensure that all samples met the quality requirements for downstream sequencing applications (Thermo Fisher Scientific, Quincy, Massachusetts, USA).

### WGS

Purified DNA was prepared for sequencing using the Oxford Nanopore Technologies (ONT) MinION platform. This technology was selected due to its portability and capacity for real-time, high-throughput genomic sequencing, which is advantageous for the rapid surveillance of microbial resistance and virulence features. Before sequencing, samples were barcoded with the Nanopore Rapid Sequencing gDNA barcoding kit SQK-RBK110.96 (Oxford Nanopore Technologies Ltd, Oxford, United Kingdom), in accordance with the manufacturer’s instructions.

### Bioinformatics analysis

Sequencing read quality (FASTQ files that contain sequence data for each read and associated per-base quality scores) generated by the MinION platform was assessed using FastQC software (Babraham Institute, Cambridge, UK), which evaluated metrics such as sequence quality, read length, and overall integrity [24]. Preliminary analyses were carried out using the ONT Epi2Me web-based tool (https://epi2me.nanoporetech.com/). Further bioinformatics processing was conducted through Galaxy Europe, a user-friendly online platform designed for accessible, reproducible, and collaborative genomic analysis that does not require command-line proficiency, thereby enhancing the reliability and transparency of results [25]. Virulence gene detection was performed using VirulenceFinder (https://cge.food.dtu.dk/services/VirulenceFinder) with stringent criteria (≥80% nucleotide identity and coverage), allowing for the precise identification of clinically significant virulence markers and emerging pathogenic strains [26]. Phylogenetic analyses were executed using RAxML (Heidelberg Institute for Theoretical Studies, Heidelberg, Germany) [27], with the final phylogenetic trees and associated heatmaps generated using iTOL [28]. The phylogenetic classification of *E. coli* isolates into respective phylogroups was conducted using the Clermont phylogenetic typing method, which offers dependable comparisons across both commensal and pathogenic lineages [29].

## RESULTS

### Sequencing quality control

The DNA sequences obtained through MinION sequencing ranged from approximately 7.5–12.0 kilobases in length, reflecting sufficient fragment sizes for comprehensive genomic analyses. The total number of bases sequenced varied from 24,200,800 to 899,274,327, with average read quality scores exceeding 8.0 and ranging between 10.6 and 12.6 (Table 1). These quality control metrics met the established thresholds for downstream analyses.

**Table 1:** Quality control assessment of sequencing data from pig slaughterhouse samples.

Sample	Quality control			

	Total bases	Mean read length	Mean quality score	The median read length
1F	331,860,026.00	8,823.20	11.1	5,610.00
2F	430,228,847.00	8,362.60	11.8	4,860.00
3F	899,274,327.00	9,990.70	12.3	6,482.00
4F	532,503,519.00	9,914.10	10.6	5,833.50
5F	150,128,479.00	8,592.00	10.5	3,624.00
6F	208,615,498.00	6,546.60	10.8	3,634.00
7F	210,908,649.00	7,639.10	11.1	3,439.00
8F	271,616,041.00	7,450.50	10.8	4,157.00
9F	411,562,364.00	8,708.80	11.4	5,175.00
10F	319,188,379.00	8,735.10	11.8	5,187.00
1E	384,759,811.00	9,152.20	12.3	5,090.00
2E	438,657,283.00	9,400.70	12.1	5,844.50
3E	400,897,053.00	9,858.00	12.1	6,023.00
4E	358,341,430.00	10,687.50	12.6	6,974.00
5E	254,046,002.00	9,184.60	12.3	6,060.00
6E	196,020,451.00	9,908.00	11.8	6,359.00
7E	24,200,800.00	11,968.70	12.5	7,923.50
8E	294,024,567.00	9,957.50	11.8	6,490.50
9E	439,363,285.00	10,272.50	12.4	6,469.00
10E	280,540,510.00	9,036.00	12.1	4,039.00

F=Floor, E=Effluent

### Virulence gene diversity and distribution by pathotype

WGS revealed the presence of 57 virulence genes among antibiotic-resistant *E. coli* isolates collected from slaughterhouse environments. These comprised 46 genes associated with enteric pathogenic *E. coli* and 15 with ExPEC. Each isolate harbored between two and 12 distinct virulence genes. Enteric pathogenic *E. coli* was detected in 45% of isolates, while ExPEC was present in 95% of isolates (Table 2).

**Table 2:** Pathotypes and virulence genes of *E. coli* in pig meat samples.

Sample type	Isolate code	Pathotypes and virulence genes					Total	
	
		Enteric pathogenic *E. coli*			ExPEC		Enteric pathogenic *E. coli* (%)	ExPEC (%)
	
		EHEC	EPEC	ETEC	UPEC	NMEC		
Floor swab	1F				*iroN, iroE, iroD, iroC,* and *iroB* are expressed as follows:			V
	2F			*etpB, etpA*			V	
	3F				*iroN, iroE, iroD, iroC, iroB, iutA, iucD, iucC, iucB, iucA*			V
	4F				*vat, iroN, iroE, iroD, iroC, iroB, iutA, iucD, iucC, iucB, iucA*			V
	5F				*iroD, iroC, iroB, iucA, iucB,* and			V
	6F				*iutA, iucD, iucA, iucB, iucC,* and			V
	7F				*iroN, iroE, iroD, iroC,* and *iroB* are expressed as follows:			V
	8F				*iroN, iroE, iroD, iroC, iroB, iutA, iucD, iucC,* and			V
	9F	*espB, espD,* and *espH*			*iroD, iroC,* and *iroB* are expressed as follows:		V	V
	10F	*gspG, gspH, gspI, gspJ,* and		gspC, gspD, gspE, gspF, and	*entD*		V	V
Effluent	1E	*espL4, fimB, fimA, fimI, fimC, fimD, fimF,* and *fimE.*	espX4, espX5		*entD*	*aslA*	V	V
	2E				*iroN, iroE, iroD, iroC,* and			V
	3E				*iroC, iroB, iucA, iucB, iucC,* and			V
	4E	*espL1, flhA, cheY, cheD, fliC, fliG, fliI, fliM*			*entD*		V	V
	5E			*etpA, etpB*	*iroN, iroE,* and *iroD:*		V	V
	6E	*espL1, espL4, espR1, espX1*			*entD, entE, entF, entS,* and		V	V
	7E	*espR1, flgJ, flgH, flgG, flgE, flgD, flgM, csgB, csgD*			*entD*		V	V
	8E				*iucD, iutA*			V
	9E				*iroN, iroE, iroD, iroC, iroB, iutA, iucD, iucC,* and			V
	10E	*espR1*			*iroN, iroE, iroD, iroC,* and *iroB* are expressed as follows:		V	V
Total number of pathotypes		*7*	1	3	19	1	9	19
Percentage of each pathotype (%)		*35*	5	15	95	5	45	95

*E. coli*=*Escherichia coli*, F=Floor, E=Effluent, EHEC=Enterohemorrhagic *E. coli*, EPEC=Enteropathogenic *E. coli*, ETEC=Enterotoxigenic *E. coli*, ExPEC=Extraintestinal pathogenic *E. coli*, UPEC=Uropathogenic *E. coli*, NMEC=Neonatal meningitis *E. coli*

Three enteric *E. coli* pathotypes were identified: EHEC, ETEC, and EPEC. In addition, two extraintestinal pathotypes were observed: UPEC and NMEC. EHEC was the most frequently detected enteric pathotype, accounting for 35% of isolates, whereas UPEC predominated among extraintestinal pathotypes at 95% prevalence (Table 2).

**Figure 2:**
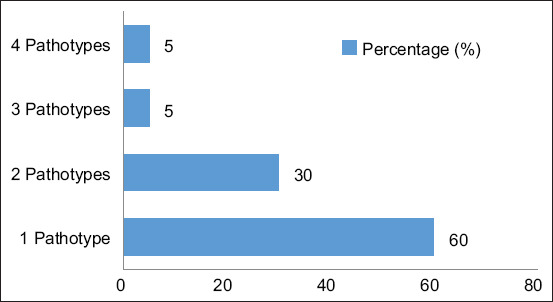
Virulence gene pathotype patterns of *Escherichia coli*. The vertical axis of the graph represents the pattern of virulence gene profiles of *E. coli* from pig slaughterhouse samples, whereas the horizontal axis shows the percentage of each virulence gene profile pattern.

The distribution of pathotypes indicated that all isolates carried virulence genes corresponding to at least one specific pathotype. A subset of isolates harbored virulence genes from up to four distinct pathotypes. The most frequent pattern observed was the presence of a single pathotype per isolate, noted in 60% of samples (Figure 2).

### Phylogroups and serotypes

**Figure 3:**
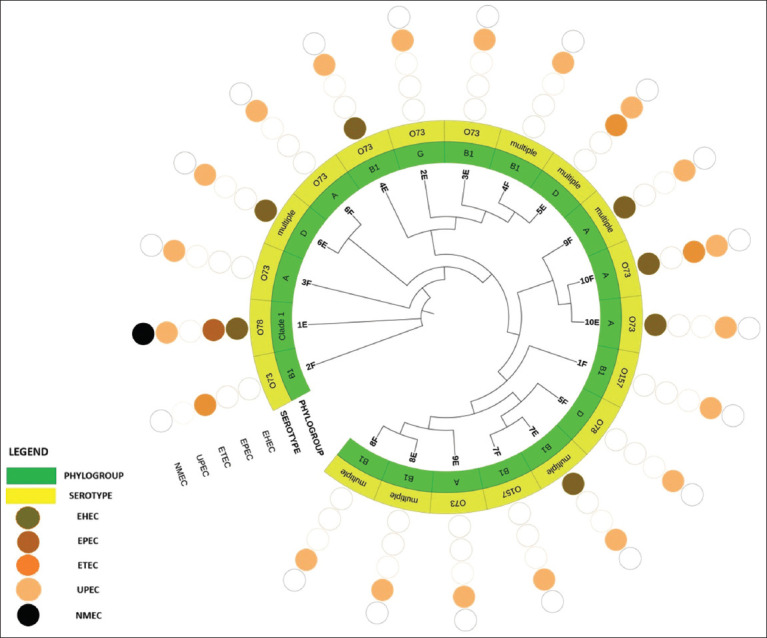
Phylogenetic relationships of *Escherichia coli* pathotypes, phylogroups, and serotypes.

Phylogenetic analysis grouped the *E. coli* isolates into five phylogroups: A, B1, D, G, and clade I, with phylogroups A and B1 being the most prevalent. Among them, phylogroup B1 accounted for the largest proportion (45%). Serotyping analysis identified four serotypes – O73, O78, O157, and mixed – of which O73 was the most dominant (Figure 3).

### Relationships among phylogenetics, pathotypes, phylogroups, and serotypes

Phylogenetic analysis of antibiotic-resistant *E. coli* isolates revealed the formation of three primary clusters. The first cluster comprised isolate 2F, the second included isolate 1E, and the third cluster was subdivided into two clades: One containing isolate 3F and the other consisting of 17 isolates. Notably, several paired isolates – such as 10F and 10E, 6E and 6F, 7E and 7F, 8E and 8F, and 4F and 5E – exhibited close phylogenetic relationships (Figure 3).

Overall, there was no evident correlation between phylogenetic clustering and the distribution of virulence gene pathotypes among antibiotic-resistant *E. coli*. Nonetheless, isolates that were closely related phylogenetically were more likely to share the same phylogroup and serotype.

## DISCUSSION

All *E. coli* isolates examined in this study possessed at least two virulence genes, indicating a baseline level of pathogenic potential. Prior research has documented that commensal *E. coli* strains isolated from healthy pigs and humans may carry virulence genes [30–32]. These genes are often considered fitness factors that enhance the survival of *E. coli* in the gastrointestinal tract and are not necessarily indicative of pathogenicity [33]. However, studies by Kaper *et al*. [34] and Pokharel *et al*. [35] suggest that a higher number and broader diversity of virulence genes are correlated with increased pathogenic potential in *E. coli* isolates.

In the present study, virulence genes commonly associated with ExPEC – particularly *iutA* and *iroN* – were predominantly identified in wastewater samples from pig slaughterhouses, with a prevalence of 95%, exceeding that of enteric pathogenic *E. coli* (45%) (Table 2). Similarly, a study by Savin *et al*. [36] conducted in Germany found that ExPEC-related genes such as *iutA* (58.3%) and *iroN* (30.6%) were prevalent in pig slaughterhouse waste. ExPEC strains are highly adaptable and capable of causing a range of extraintestinal infections including UTIs, sepsis, prostatitis, pneumonia, and meningitis in both humans and animals. Among these, UPEC is responsible for up to 90% of UTIs cases in humans and animals [37]. Comparable virulence gene profiles and serogroups have been identified in ExPEC isolates from pigs and humans, indicating a potential for zoonotic transmission and cross-species infection [38, 39]. Consequently, ExPEC strains originating from pigs pose serious risks to both food safety and public health [40, 41].

In pigs, *E. coli* infections are primarily associated with colibacillosis, a disease that manifests in several clinical forms such as neonatal diarrhea, post-weaning diarrhea, edema disease, septicemia, polyserositis, coliform mastitis, and UTIs [42]. The ETEC, EHEC, and EPEC pathotypes are recognized as the principal causes of severe clinical manifestations and disease outbreaks throughout various stages of pig production [8]. Virulence genes linked to all three of these pathotypes were also detected in this study (Table 2). For example, Kagambèga *et al*. [43] found EHEC and EPEC to be dominant among *E. coli* strains in pigs slaughtered in Ouagadougou, Burkina Faso. Our findings similarly show that EHEC was the predominant pathotype (35%), highlighting its critical role in foodborne outbreaks and the urgent need for targeted monitoring and prevention measures. ETEC and EPEC followed at 15% and 5%, respectively.

In this study, EHEC-associated virulence genes were largely composed of effector class genes such as *espR1*, *espL1*, *espL4*, *espX1*, and *espH* (Table 2). These genes encode components of the Type III Secretion System (T3SS), which enables the translocation of effector proteins into host cells and contributes to the formation of characteristic attachment and effacing lesions [44]. EHEC has been implicated in severe clinical outcomes in both humans and animals, including extraintestinal complications like hemolytic uremic syndrome [45]. Transmission often occurs through contaminated food or water, and pigs are considered significant reservoirs. Pork products have been associated with multiple EHEC-related outbreaks [8, 10], with serotype O157:H7 representing a major public health concern globally. EHEC outbreaks linked to pork consumption have been reported in Japan, several European countries, and North America [46–49].

ETEC was the second most prevalent pathotype identified in this study. ETEC is a known cause of acute diarrhea in piglets [50] and is also responsible for traveler’s diarrhea and pediatric diarrhea in developing countries, with some cases resulting in death. According to the WHO, ETEC is responsible for over 157,000 Diarrhea-related deaths annually, particularly affecting children under 2 years of age [51]. The principal virulence factors of ETEC include colonization factors and enterotoxins [52]. In this study, the key colonization-related virulence genes detected were *etpA* and *etpB* (Table 2). These genes facilitate bacterial adherence to host epithelial cells by interacting with conserved flagellar regions through extended appendages (<10–15 mm) and binding to adhesins such as *etpA*. Flagellar structures play an essential role in the initial adhesion, colonization, and pathogenesis of ETEC [53]. Furthermore, *etpA* is a secreted autotransporter glycoprotein that contains N-acetylgalactosamine, a sugar also found in blood group A antigens, which may contribute to heightened disease severity in individuals with blood group A [54].

ETEC-induced enteric colibacillosis in pigs is associated with substantial morbidity and mortality, with reported mortality rates reaching as high as 70% in neonatal piglets suffering from acute watery diarrhea [42, 55]. ETEC can be transmitted through contaminated feed, water, soil, or environmental surfaces in pig farming facilities. Its prevalence varies significantly by region, ranging from 86.5% in Spain and 72.0% in South Africa, to as low as 15.2% in Argentina [9, 56]. The spread of virulence genes among *E. coli* isolates is facilitated by horizontal gene transfer, often mediated through plasmids and pathogenicity-associated islands. These mobile genetic elements frequently carry multiple virulence genes, including those encoding the T3SS (e.g., *esp*) and iron acquisition systems such as *iutA* and *iroN* [56, 57].

Phylogenetic analysis in this study demonstrated close genetic relationships between isolates derived from floor swabs and effluent samples collected at the same slaughterhouses – specifically, isolates 10F/10E, 6E/6F, 7E/7F, and 8E/8F (Figure 3). This suggests potential transmission of antibiotic-resistant *E. coli* from infected pigs to the slaughterhouse environment, with subsequent dissemination into effluent, thereby posing a risk to surrounding ecosystems. No clear association was observed between phylogenetic proximity and the virulence gene profiles of the isolates (Figure 3). This finding may be attributable to diverse sources of contamination within slaughterhouses, including inadequate worker hygiene and substandard waste management practices.

The predominance of phylogroups B1 and A – generally regarded as commensal or weakly pathogenic – raises concerns about potential transitions from benign to pathogenic forms, particularly in the context of zoonotic transmission (Figure 3). While these phylogroups are commonly linked with commensal *E. coli*, they are also associated with enteric and extraintestinal pathotypes in pigs [58, 59]. In fact, their involvement in ExPEC infections among pigs has been well documented [60]. Serotyping in this study identified four serotypes – O73, O78, O157, and mixed – with O73 being the most prevalent (Figure 3). Although serotype O73 is typically considered non-pathogenic, some strains have been implicated in ExPEC-related UTIs [61]. Conversely, O157 and O78 are well-recognized pathogenic serotypes in both humans and animals, depending on the presence of specific virulence determinants [62].

## CONCLUSION

This study provides critical insights into the phylogenetic diversity, virulence gene profiles, and pathotypes of antibiotic-resistant *E. coli* isolates from pig slaughterhouses in Banten Province, Indonesia. Using WGS, we identified five distinct phylogroups (A, B1, D, G, and clade I), with phylogroups A and B1 predominating. Importantly, a high prevalence of virulence genes was documented, predominantly associated with the ExPEC, particularly UPEC (95%), indicating significant zoonotic risks. Enteric pathogenic strains (EHEC, ETEC, and EPEC) were also identified, with EHEC being the most prevalent.

The detection of diverse virulence gene profiles and MDR phenotypes highlights the considerable public health threats posed by the pig slaughterhouse environment, with potential implications for human infections, food safety, and environmental contamination. Furthermore, close phylogenetic relationships between isolates from different sample types (floor swabs and effluents) suggest that ongoing transmission events are facilitated by inadequate hygiene practices and poor waste management at slaughterhouses.

Despite these contributions, this study has certain limitations. This method primarily analyzed isolates collected at a single time point, limiting the assessment of temporal dynamics and persistence of pathogenic strains in slaughterhouse environments. Furthermore, although WGS technology provided comprehensive genomic data, the functional expression of the detected virulence genes was not examined, which would require additional functional studies.

To address these limitations, future research should incorporate longitudinal surveillance studies to better understand the dynamics of resistant and pathogenic strains over time. In addition, further research should investigate the mechanisms underly-ing the transfer and expression of virulence and resistance genes in environmental and animal reservoirs. Ultimately, this study underscores the urgent need for improved hygiene standards, stricter antimicrobial stewardship in pig farming, and routine genomic surveillance to mitigate public health risks posed by antibiotic-resistant *E. coli* in Indonesia and globally.

## AUTHORS’ CONTRIBUTIONS

HL, DFP, CB, and DI: Statistical analysis, interpretation of results, and drafted, reviewed, and edited the manuscript. DFP: Sample collection. HL: Managed the study. PR and DFP: Laboratory analysis and data curation. HL, CB, and IWTW: Conceptualization and study management. HL and CB: Conceptualized the study, implemented the study, and supervised the field and laboratory works. All authors have read and approved the final manuscript.
